# Functional assessment of the *BMPR2* gene in lymphoblastoid cell lines from Graves’ disease patients

**DOI:** 10.1111/jcmm.13425

**Published:** 2017-12-20

**Authors:** Guillermo Pousada, Mauro Lago‐Docampo, Sonia Prado, Rubén Varela‐Calviño, Beatriz Mantiñán, Diana Valverde

**Affiliations:** ^1^ Department of Biochemistry, Genetics and Immunology Faculty of Biology University of Vigo Vigo Pontevedra Spain; ^2^ Instituto de Investigación Biomédica de Ourense‐Pontevedra‐Vigo Pontevedra Spain; ^3^ Department of Biochemistry and Molecular Biology University of Santiago de Compostela A Coruña Spain; ^4^ Endocrine, Diabetes, Nutrition and Metabolism Department Complexo Hospitalario Universitario de Vigo Pontevedra Spain

**Keywords:** pulmonary arterial hypertension, Graves’ disease, BMPR2, functional analysis, lymphoblastoid cell lines, Epstein–Barr virus, expression assay, TGF‐β/BMP

## Abstract

In this study, we analysed the possible influence of the c.419‐43delT *BMPR2* variant in patients with Graves’ disease (GD), in a molecular basis, focusing our efforts on possible alterations in the mRNA processing and synthesis. The molecular assessment of this variant in patients with GD would shed light on the association between the *BMPR2* gene and the disease. The variant was detected in 18%, 55% and 10% of patients with pulmonary arterial hypertension, GD and in general population, respectively. Patients with GD fold change showed increased *BMPR2* expression when matched against the controls, with a mean of 4.21 ± 1.73 (*P* = 0.001); *BMPR2* was overexpressed in the analysed cell cycle stages. Fold change analysis of variant carriers and non‐carriers showed slight overexpression and differences between phases, but none of them were statistically significant. BMPR2 expression was confirmed in the lymphoblastoid cell lines (LCLs) with a molecular weight of 115 kD, and no differences between variant carriers and non‐carriers were detected. To conclude, the *BMPR2* variant c.419‐19delT appears in high frequency in patients with GD, and independently of its presence, BMPR2 is overexpressed in the LCLs from the GD patients tested. This increase could be paired with the described decreased expression of transforming growth factor‐β1 in thyroid tissue from patients with GD.

## Introduction

The *BMPR2* gene (OMIM #600799) is located in the chromosome 2 position q33, and it encodes for the bone morphogenetic protein receptor of type 2 (UNIPROT #Q13873), a constitutively active serine/threonine kinase receptor that plays a major role in the transforming growth factor beta (TGF‐β; UNIPROT #P01137) superfamily regulation 1, 2. Mutations in the germinal line of BMPR2 have been widely related to at least 50% of the familial cases and 40% of the idiopathic of pulmonary arterial hypertension (PAH; OMIM #178600, ORPHA 422) 1, 3, 4, 5, 6. Although PAH shows a reduced penetrance pattern, only 20% of the mutation carriers end up developing the disease 7.

Pulmonary arterial hypertension is a rare disease characterized by precapillary pulmonary arteries remodel resulting in loss of permeability and increased blood flow resistance that eventually leads to right heart failure 8, 9, 10. PAH has an annual incidence between 1 and 2 cases per million 11, and in the Spanish population, it has an incidence between 1.2 and 3.2 and a prevalence between 4.6 and 16 cases per million 12, affecting between two and four times more women than men 13, 14. Average age of diagnosis is about 45 years 15, even though symptoms may appear at any age 8. PAH can be classified as idiopathic (IPAH), familial (FPAH) or associated with other diseases such as connective tissue diseases, congenital heart diseases, portal hypertension and drug or toxin exposure (APAH) 9, 14. Besides, several cases of associated PAH to GD have been reported 16, 17, 18.

After *BMPR2* screening in PAH patients, we detected the c.419‐43delT mutation in several of them 5. Whenever we start to study a new mutation, a population analysis is carried out using DNA samples from general population and patients that have given their consent for the usage and storage of their anonymized genetic material by our group in future projects. We found this variant with a frequency up to a 40% in a series of samples from an investigation project on thyroid autoimmunity from the Endocrinology Service of the Complexo Hospitalario Universitario de Vigo.

These findings lead us to analyse the whole set of samples from that project which consisted in GD patients samples. GD is one of the most common autoimmune disorders, with an annual incidence of approximately 21 new cases per 100,000 individuals 19, affecting mostly women with a 5:1 ratio 20. Clinical presentation of the GD is characterized by thyrotoxicosis, goitre and ophthalmopathy. Although the only common sign between every patient is the hyperthyroidism 21 caused by antibodies that imitate the action of the thyroid‐stimulant hormone (TSH; UNIPROT #P01222) activating its receptor. As an autoimmune disorder, GD has a variety of associated pathologies, being the APAH diagnosed in about a 15% of the cardiopathies caused by GD 16, 18.

Genetic variants are constantly reported, relying on *in silico* analysis for the prediction of its pathogenicity. These kind of predictions often differ from reality, where missense, synonymous or intronic mutations can end up playing an important role in the development of a disease. Synonymous and non‐synonymous can alter protein structure, the splicing process and mRNA stability and conformation. There are several examples in the literature showing pathogenic mutations where the *in silico* analysis came back negative 3, 7, 22. The functional studies are of great value to investigate the role of these mutations in the development of a disease.

The objectives of this work were to assess the presence of the c.419‐43delT variation in patients with GD and to analyse the functionality of the mutation by minigene assay for splicing alterations and quantitative real‐time PCR for expression measurement. Functional analysis of this frequent polymorphism in patients with GD would shed light on the relationship between the *BMPR2* gene and the disease.

## Patients and methods

### Patients with GD and samples

Graves’ disease patients’ diagnosis was performed based in the laboratory examination of hyperthyroidism patients showing low or suppressed TSH and high levels of T3I and T4I. In cases where GD appeared associated with ophthalmopathy and diffuse goitre, more tests were needed. On the contrary, complementary tests depending on the centre of diagnosis were performed, such as autoantibodies against TSH receptor detection (thyroid‐stimulating immunoglobulin), thyroid scintigraphy or Doppler echography. Patients and controls signed an informed consent and the Autonomic Ethics Committee approved the study (Galician Ethical Committee for Clinical Research; *Comité Autonómico de Ética da Investigación de Galicia—CAEI de Galicia*) followed the clinical‐ethical practices of the Spanish Government and the Helsinki Declaration.

### Mutational analysis in *BMPR2* gene

Genomic DNA was extracted, using the FlexiGene DNA Kit (Qiagen, Hilden, Germany), according to manufacturer's protocol, from peripheral blood. Genomic DNA amplification was performed with 50 ng of genomic DNA from each individual with a polymerase chain reaction (PCR), using GoTaq^®^ Green Master Mix (Promega Corporation, Madison, WI, USA). The primers used to amplify the *BMPR2* gene were as described by Deng *et al*. 23, and changes in other regions were not analysed. PCR amplification was performed at 55°C annealing temperature for the exons 1, 3, 5, 6, 7, 8, 9, 10, 11 and 13 and 60°C for the exons 2 and 12. PCR products were separated by electrophoresis through 2% agarose gels containing SYBR Safe DNA Gel Stain (Invitrogen, Carlsbad, CA, USA); to confirm the PCR products, HyperLadder IV (New England Biolabs, Ipswich, MA, USA) was used as the molecular weight marker; and PCR products were purified using ExoSAP‐IT kit (USB Corporation, Cleveland, OH, USA). Sequencing reactions were performed using the BigDye Terminator version 3.1 Cycle Sequencing Kit (Applied Biosystems, Carlsbad, CA, USA), and the fragments were purified using Agencourt CleanSEQ‐Dye Terminator Removal (Beckman Coulter, Brea, CA, USA). A second independent PCR and sequencing reaction in both the forward and reverse strands was performed to check for the detected mutations.

Sequences were aligned to the reference Ensembl DNA sequence [ENST00000374580]. To predict whether changes could affect, create or eliminate donor/acceptor splice sites were used Neural Network SPLICE (NNSplice) 0.9 version from the Berkeley Drosophila Genome Project (Lawrence Berkeley National Laboratory, Berkeley, CA, USA), NetGene2 (Technical University of Denmark, Kongens Lyngby, Denmark), Splice View, Human Splicing Finder 2.4.1 version (Aix Marseille Université, Marseille, France) and Automated Splice Site Exon Definition Server‐ASSEDA (University of Western Ontario, London, Canada).

### Minigene assay

For the c.419delT variant, we amplified the exon and 250 bp of 5′and 3′ intronic junctions from the control and the GD patient DNA with high‐fidelity Phusion polymerase (Finnzymes, Espoo, Finland) to obtain the wild‐type, with primers carrying restriction sites for XhoI/NheI (New England Biolabs). The forward and reverse primers were 5′‐CTCGAGACTTGGTGTTTTAGTGTTCC‐3′ and 5′‐GCTAGCGAAAGGGGTAGTGACTGATAA‐3′, respectively. The amplified fragments were cloned using the T4 DNA ligase concentrated (New England Biolabs) in the Exon Trapping Expression Vector p.SPL3 (Invitrogen, San Diego, CA, USA), provided by Dr. José María Millán. Wild‐type and mutant construct were amplified using the GoTaq^®^ Green Master Mix (Promega Corporation) and were sequenced using the BigDye^®^ Terminator v3.1 Cycle Sequencing Kit (Applied Biosystems). The forward and reverse primers were 5′‐GCCAGGACATGGACCGCACGCTCGA‐3′ and 5′‐TCGAGCGTGCGGTCCATGTCCTGGC‐3′, respectively.

As described previously 22, 2 × 10^5^ COS‐7 cells (from kidney of *Cercopithecus aethiops*, provided by Dr. José María Millán) were grown to 90% confluency in Dulbecco's modified Eagle medium‐DMEM (Gibco, Grand Island, NY, USA) supplemented with 10% foetal bovine serum (PAA Laboratories, Pasching, Austria) and l‐glutamine penicillin/streptomycin (Gibco^®^) at 37°C and 5% CO_2_ atmosphere. COS‐7 cells were transiently transfected in triplicates with the minigene constructs using Lipofectamine 2000 reagents (Thermo Scientific, Carlsbad, CA, USA) according to manufacturer's instructions. RNA was extracted and purified using the Nucleic Acid and Protein Purification kit (NucleoSpin RNA II; Macherey‐Nagel, Düren, Germany), and cDNA was synthesized using the GeneAmp Gold RNA PCR Core Kit (Applied Biosystems). Primers used to amplify the cDNA were 5′‐TCTGAGTCACCTGGACAACC‐3′ and 5′‐ATCTCAGTGGTATTTGTGAGC‐3′, using the high‐fidelity Phusion polymerase (Finnzymes). PCR products were analysed in 2% agarose gels containing SYBR Safe DNA Gel Stain (Invitrogen) and, finally, by direct sequencing in both senses.

### Generation of LCLs by infection with Epstein–Barr virus

To obtain enough cells for gene expression from both controls and patients, immortalization of B cells and generation of LCLs was performed by infection with Epstein–Barr virus (EBV)‐containing culture media, obtained from exponentially growing tetradecanoyl phorbol acetate‐stimulated B95‐8 cells (Prof. Mark Peakman, Department of Immunobiology, King's College School of Medicine). Supernatants were stored at −20°C until usage.

To obtain LCLs, peripheral blood mononuclear cells (PBMCs) from both controls (*n* = 8) and GD patients (*n* = 7) were cultured in 25‐cm^2^ tissue culture flasks, treated for 1 hr with FK‐506 (Tacrolimus; Sigma‐Aldrich, Madrid, Spain) to inhibit the expansion of T lymphocytes and infected with EBV‐containing culture media. After 5–7 days, clusters of transformed cells were detected, and culture media were changed every 2–3 days since then as required until enough cells were available for both cell characterization and gene expression analysis. Cellular origin of the growing cells was determined by cell staining using anti‐CD19 antibodies (BD Biosciences, Madrid, Spain) and flow cytometry analysis (BD FACScalibur, BD Biosciences, Madrid, Spain). Phenotypic analysis was performed by duplicates using cells obtained 5–7 days apart, to analyse the stability of the phenotype. CD19 expression was performed after gating cells in the forward‐scatter lymphocyte region.

### Sorter and collected cells

Cells from the controls and patients with GD were cultured in a medium consisting in RPMI 1640 Medium (Gibco, Carlsbad, CA, USA). Cultures were stained with Hoechst 33342 (BD Biosciences) following the manufacturer's recommendations. Thereafter, cells were harvested and resuspended at a concentration of 1 × 10^6^/ml in sort buffer + 2 mM EDTA and DNAse and then sorted base on the DNA content. Cells in G_0_/G_1_, S and G_2_/M were collected using a BD Biosciences FACS Aria III flow cytometer (BD Biosciences, Madrid, Spain). BD FACSDiva v8.0.1 (BD Biosciences, Madrid, Spain) and Flowjo v7.6.5 (FlowJo, LLC, Ashland, OR, USA) were used to data analysis. Then, the recollected cells were resuspended in RNAlater solution from Ambion (Invitrogen) and were stored at −80°C.

### cDNA synthesis and expression assay by quantitative real‐time PCR

A total of 2 μg of total RNA was used to perform the cDNA synthesis with the GeneAmp RNA PCR Core Kit (Life Technologies, Carlsbad, CA, USA). The cDNA was later used to amplify the target gene (*BMPR2*) and the reference genes (*GADPH* and *ACTB*) by quantitative real‐time (qPCR) using commercial Taqman probes, with 4331182, 4331182 and 4331182 references, respectively (Life Technologies). The reaction was carried out in a Step One thermocycler (Life Technologies) with a total amount of 50 ng of cDNA in 20 μl, containing 10 μl of Taqman Universal PCR Mastermix (Life Technologies) and 1 μl of Taqman Gene Expression Mix (Life Technologies). The amplification conditions were as follows: 50°C for 2 min., 95°C for 10 min. and 40 cycles of 15 sec. at 95°C and 1 min. at 60°C. Expression data normalization was carried out using the Hellemans 2007 method 24 to calculate the normalized relative quantities (NRQ) and the fold change for the intergroup comparison.

### Confirmation of the BMPR2 Protein expression by Western blot

Western blot analysis was performed using 20 μg of protein obtained from LCLs and cultured B lymphocytes. Laemmli sample buffer (Bio‐Rad, Hercules, CA, USA) containing 5% β‐mercaptoethanol was used to dilute the samples before heating them at 95°C for 5 min. Proteins were separated by SDS‐PAGE using a 7.5% TGX Mini‐Protean precast gel (Bio‐Rad). After electrophoresis, protein transfer into a PVDF membrane was performed using the Transfer Blot Turbo Transfer Pack (Bio‐Rad) in a semidry blotting apparatus (Bio‐Rad) for 7 min. at 1.3 A. The membrane was then blocked for 1 hr at room temperature using 5% non‐fat milk in Tris‐buffered saline (TBS) containing 0.1% Tween 20 (TBST). The immunoblotting was performed incubating the membrane overnight at 4°C with mouse monoclonal anti‐BMPR2 IgG (reference Ab130206; ABCAM, Cambridge, UK) diluted 1/250 in blocking solution. After washing with blocking solution for 3 times, the membrane was incubated with 1/15,000 dilution of rabbit antimouse IgG conjugated with horseradish peroxidase (reference A9044; Sigma‐Aldrich, St. Louis, MO, USA) for 45 min. in blocking solution. TBS was then used to wash the membrane.

BMPR2 was detected by chemiluminescence using the Clarity Western ECL Substrate (Bio‐Rad) in a ChemiDoc™ (Bio‐Rad) digital camera‐based imaging system. Loading control was performed by Coomassie Brilliant Blue (R‐250) staining as described by Welinder and Ekblad in 2010 25.

### Statistical analysis

Statistical analysis was carried out using the IBM‐SPSS v.19 software (IBM, Armonk, NY, USA) and GraphPad Prism (GraphPad Software INC, La Jolla, CA, USA). Nonparametric statistic tests were used for intragroup analysis (Kruskal–Wallis and Wilcoxon) and intergroup (Mann–Whitney). Probability values of less than 0.05 were considered statistically significant.

## Results

### Controls and GD patient samples

Eight controls, obtained from the Faculty of pharmacy of University of Santiago de Compostela, seven patients with GD, obtained from the Endocrine, Diabetes, Nutrition and Metabolism Department of Complexo Hospitalario Universitario de Vigo and 100 individuals from general population, obtained from Complexo Hospitalatio Universitario de Vigo, were included in this analysis. Four of the controls were women, and four were men; their medical history and analytics showed that all values were within normal parameters, and no chronic pathologies nor hyperthyroidism/hypothyroidism were detected either. However, all patients with GD included in this study were women. The clinical characteristics of GD patients are summarized in Table 1.

**Table 1:** Clinical features of GD patients included in this study

GD patients	Gender	Age at diagnosis	Ophthalmopathy	Goitre	TSI	Thyroid scintigraphy	Doppler echography
1	Female	31	Positive	Diffuse	Positive	Hypercaptation	ND
2	Female	53	Negative	Diffuse	Positive	Hypercaptation	ND
3	Female	33	Positive	Diffuse	Positive	Hypercaptation	ND
4	Female	42	Negative	Diffuse	Negative	Hypercaptation	ND
5	Female	73	Negative	Nodular	Positive	ND	ND
6	Female	56	Positive	Diffuse	Positive	ND	ND
7	Female	72	Negative	Nodular	Positive	Hypercaptation	ND

GD, Graves’ disease; TSI, thyroid‐stimulating immunoglobulin; ND, not determined.

For this analysis, we have differentiated between individuals from general population and controls. The first group represents random Galician population that did not have their medical history checked; their samples were only used to assess the frequency of the polymorphism in population from the same area as the patients. While, as stated above, controls were tested to assure the absence of health conditions that could jeopardize the results.

### Mutational frequency of the *BMPR2* gene in controls, GD patients and general population

**Figure 1:**
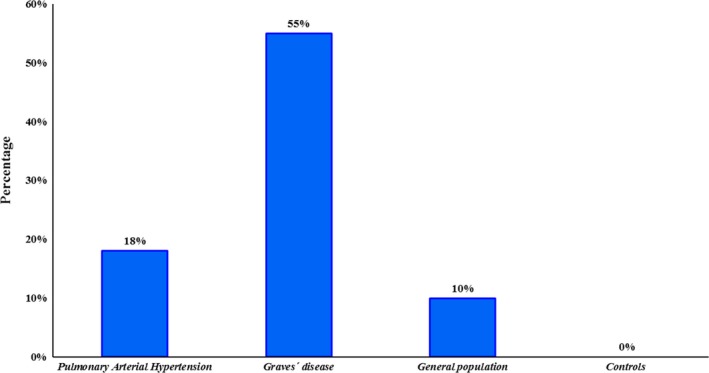
Percentage of the c.419‐43delT variant in pulmonary arterial hypertension (PAH), Graves’ disease, general population and controls. An 18% of PAH patients carried this deletion and a 55% of patients with Graves’ disease carried this variant too. However, the variant was only present in a 10% of general population and none of our controls carried it.

We checked c.419‐43delT variant, in *BMPR2* gene, in 57 patients with PAH, in 40 patients with GD and in 100 individuals from general population, found the deletion in 18% of patients with PAH, in 55% of patients with GD and in 10% of individuals from general population (Fig. 1). Moreover, after the mutational analysis of *BMPR2* gene in the controls we did not find variants in exons and intronic junctions nor in 5′UTR region. However, in three the seven analysed GD patients we identified the c.419‐43delT variant.

**Figure 2:**
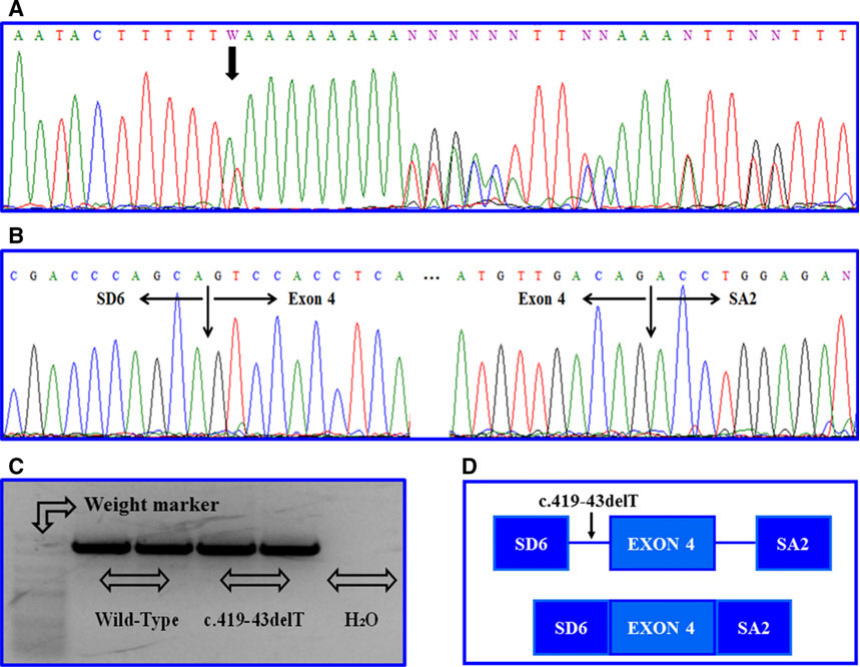
Functional analysis of c.419‐43delT variant by minigene assay. (**A**) Representative sequence electropherograms for the c.419‐43delT variant in *BMPR2* gene. (**B**) Sequencing results for *in vitro* mRNA processing for c.419‐43delT variant. (**C**) Agarose gel electrophoresis shows the band pattern of the transcripts obtained after mRNA processing for c.419‐43delT variant. (**D**) Representative mRNA processing for c.419‐43delT variant. The variant produced unchanged splicing when comparing wild‐type and mutant.

After the *in silico* analysis of c.419‐43delT, the splicing tools had foretold the benign nature of this deletion. Besides, we did not find alterations in the RNA processing using the minigene assay for the c.419‐43delT variant in *BMPR2* gene in comparison with the wild‐type sequence, and the results showed that this deletion did not generate a new transcript (Fig. 2). In addition, we checked the splicing process using RNA from LCLs and the results were the same as in the minigene assay.

### Phenotypic characterization of LCLs

Growing cells were characterized by flow cytometry after staining with anti‐CD19 monoclonal antibodies. All growing LCLs obtained from both controls and GD patients were CD19‐positive confirming their B‐cell origin. LCLs obtained from controls were almost all CD19 positive, and this percentage remained stable with time (97.2 ± 1.4% in the first analysis and 97.7 ± 1.5% in the second analysis) (mean ± SD); when a similar procedure was carried out using PBMCs obtained from patients with GD, very similar results were obtained compared to those from controls (95.6 ± 2.1 in the first analysis and 95.3 ± 2.2 in the second analysis) (mean ± SD). These data confirm the B‐cell origin of the growing cell lines obtained from both controls and GD patients and also the stability of the cell phenotype with time.

Cell separation went as expected, obtaining a higher number of cells for the G_0_/G_1_ phase. After sorting the LCLs using the BD Biosciences FACS Aria III flow cytometer (BD Biosciences). For the control samples, 500,000 cells were isolated for the G_0_/G_1_ phase and S phase and 100,000 cells for the G_2_/M phase. Same numbers were obtained for the patients group, with GD, for G_2_/M phase, and were isolated 250,000 cells for G_0_/G_1_ and S phases.

### Expression assay of the *BMPR2* gene in LCLs

**Figure 3:**
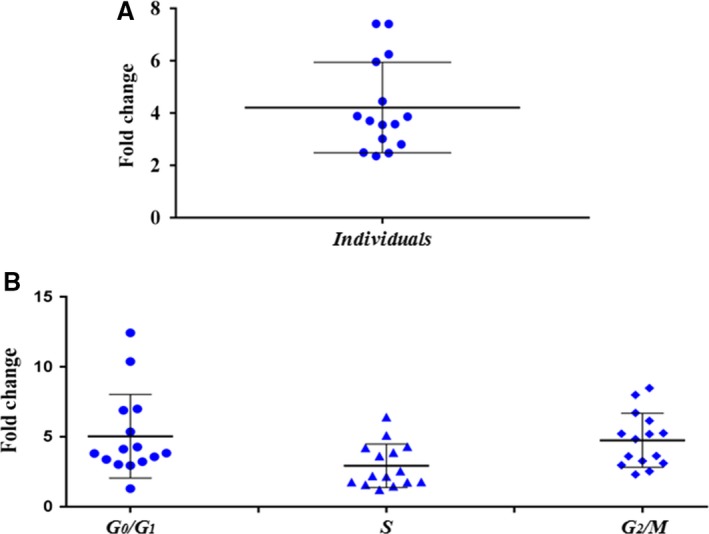
Expression assay results of Graves’ disease (GD) patients and controls. (**A**) Dot plot representing the fold change between GD patients and controls without taking into account cell cycle stage. The data showed augmented expression for the patients with GD when compared against the controls, and these data are statistically significant (*P* = 0.001). (**B**) Dot plot representing the fold change between GD patients and controls taking into consideration cell cycle stage. The data showed increased gene expression for the patients in all the cell cycle stages, and there were no statistically significant differences between stages.

*BMPR2* gene expression for patients with GD was detected with a mean threshold cycle (CT) value of 34.38 ± 2.04 (mean ± SD). In controls, the mean CT was 34.05 ± 0.94. NRQ values for controls showed similar expression levels between G_0_/G_1_ and S with a slight decrease in G_2_/M. However, patients with GD G_0_/G_1_ and G_2_/M phases had similar values, while S phase showed a noticeable drop. These intragroup differences were not statistical significant. The fold change obtained after comparing the controls and the patients with GD showed a generalized increase of expression for the patients (Fig. 3A), each dot representing individual data compared against the opposite group (controls and GD patients), showing a mean value of 4.21 ± 1.73 and a median value of 3.71. The increased *BMPR2* expression was detected in all the analysed cell cycle phases (Fig. 3B), and G_0_/G_1_ and G_2_/M phases showed mean fold values of 5.03 ± 3 and 4.75 ± 1.94, and median values of 3.84 and 4.83, respectively, S phase showed a mean fold increase of 2.93 ± 1.56 and a median of 2.19. These intergroup differences are statistically significant (*P* = 0.001).

**Figure 4:**
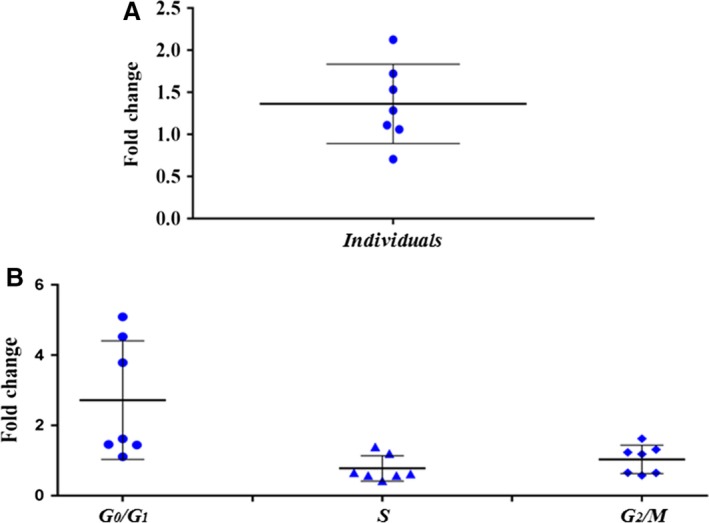
Expression assay results of c.419‐43delT carriers and wild‐type patients. (**A**) Dot plot representing the fold change between carriers and wild‐type patients without taking into consideration cell cycle stage. The results showed a slight increase in the variant carriers’ *BMPR2* expression, and these data were not statistically significant. (**B**) Dot plot representing the fold change between variant carriers and wild‐type patients taking into account cell cycle stage. Data showed a rise of gene expression in the G_0_/G_1_ and a slight decrease for S phase for the carriers, while in the G_2_/M, it showed almost identical expression between both groups. There were no statistically significant differences between stages.

The GD patients group can be spliced between wild‐type and polymorphism carriers, and polymorphism carriers showed a mean CT of 34.6 ± 1.99 for *BMPR2* detection while the wild‐type had a value of 34.08 ± 2.16. NRQ values for wild‐type showed the lowest expression levels in the G_0_/G_1_ phase, an slight increase in S, and its maximum levels in the G_2_/M. Carriers showed slightly superior NRQ values than the WT in all phases, with similar levels in the G_0_/G_1_ and S phases and a slight increase in the G_2_/M, these differences were not statistical significant. The fold change obtained from these groups comparison resulted in greater expression for carriers (Fig. 4A), with a mean value of 1.36 ± 0.47 and a median of 1.28. This slight overexpression showed greater fold levels in the G_0_/G_1_ phase with a mean value of 2.72 ± 1.69 and a median of 1.62, a slight decrease in the S phase with 0.78 ± 0.36 mean value and a median of 0.62; and equal levels for G_2_/M with mean values of 1.03 ± 0.41 (Fig. 4B) and median of 1.18.

### Expression confirmation of the BMPR2 protein by Western blot assay in LCLs

**Figure 5:**
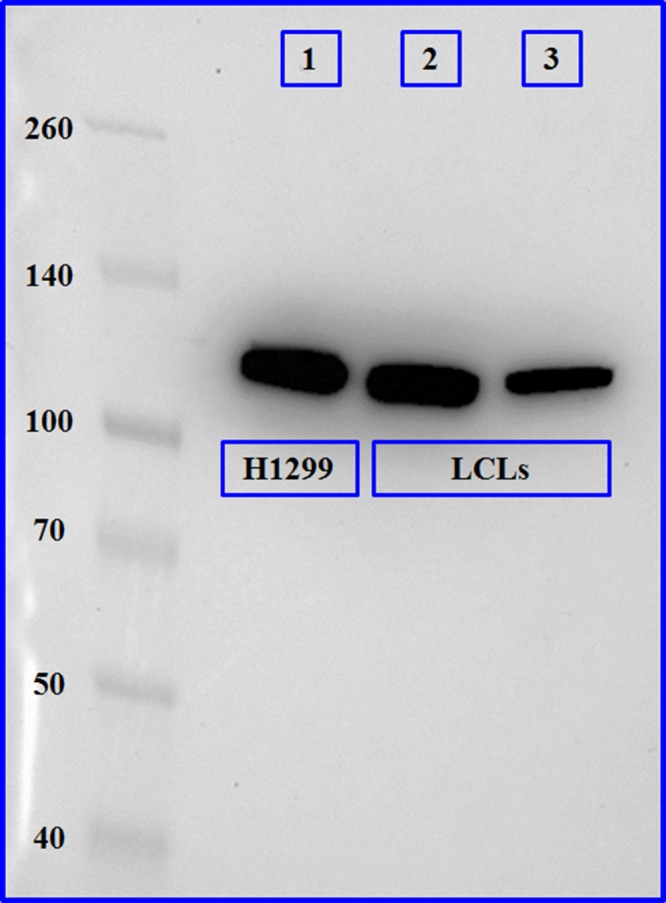
Western blot results for BMPR2. Expression of the BMPR2 protein was confirmed in both, variant carriers (lane 2) and non‐carriers (lane 3), to confirm the qPCR results and assess that the protein molecular weight was correct, no further measurements were performed.

BMPR2 was detected in the positive control (H1299 cell line, lane 1) and the lymphoblastoids samples matching its predicted molecular weight of 115 kD, both in the c.419‐43delT (lane 2) and the wild‐type sample (lane 3). These results are shown in Figure 5.

## Discussion

The advances in mutations’ detection methods offer us high amounts of data about the changes that we can find in the population, although there are not yet reliable systems that allow the determination of mutations’ pathogenicity nor of those variants that may have an uncertain significance 26. Thus, when a SNP is detected in high frequency in specific populations, pathology linkage should be considered, taking into account the enormous rate of variant's reclassification 27. Assessing the functionality of the identified mutations is especially important in rare diseases, where it has gained relevance in the last years, as it enhances our ability to correctly identify pathologies or even gives us the possibility of predicting something as valuable as patient's prognosis. Therefore, functional characterization of gene variants reduces the difficulty of correctly cataloging them 28, 29 and helps in the task of creating more accurate bioinformatic applications.

The c.419‐43delT variant is relatively common in the European population, where it has a frequency of 12.7% by ExAc and an 11.7% by Ensembl that when subdivided into the Iberian population lowers to an 8.4%. In our case, this variant was found in 18% of the PAH patients analysed, a proportion bigger than the expected after checking the SNPs databases, but this data were diminished after we found the polymorphism in 55% of the GD patients analysed. These data may indicate that the variant in study displays higher implication than expected in both PAH and GD that was still unknown. In addition, our analysis of the c.419‐43delT variant in controls and general population supported the described frequencies in different databases.

In this study, we hypothesize that the variant c.419‐43delT, classified as a polymorphism after an exhaustive *in silico* analysis, might have an influence in patients diagnosed with GD. Schleinitz *et al*. had only previously studied this variant in a cohort, where they analysed the role of *BMPR2* gene in the pathophysiology of obesity, and no genotype/phenotype correlation was established for this variant 30. The high frequency found in our study lead us to believe about a possible linkage with GD. GD is defined as a multifactorial disorder, being caused by a complex interaction between genotype and environment, which would lead to the loss of tolerance against the thyroid antigens, and therefore, being the first step of immune response against the thyroid gland 21. In several families with recurrent autoimmune thyroiditis, there has been described a positive linkage between GD and various genes that can be divided among immune system regulators (*human leukocyte antigen—antigen D regulated*,* HLA‐DR*;* cytotoxic T‐lymphocyte‐associated protein4*,* CTLA4*–OMIM #123890; *cluster of differentiation 40*,* CD40*—OMIM #109535; and *protein tyrosine phosphatase non‐receptor type 22*,* PTPN22*—OMIM #600716) and thyroid specific (*thyroglobulin*,* TG*—OMIM #188450; and *thyroid‐stimulating hormone receptor*,* TSHR*–OMIM #603372) had been established, showing reduced penetrance 31.

After performing the minigene assay for the c.419‐43delT variant to verify its pathogenicity *in vitro*, we did not find any differences concerning transcript processing between wild‐type and mutant constructions, as this variant is not located in a consensus splicing region 32. Likewise, we performed functional studies, using lymphoblastoid mRNA from carrier patients and patients without the deletion to confirm the minigene assay results. The mRNA analysis using lymphoblastoids did not detect any change in the splicing process. However, the splicing effect is tissue dependent; thus, these results should be taken carefully as they have to be confirmed using vascular endothelial cells or thyroid gland cells 22.

To analyse the expression pattern, we performed qPCR analysis in PBMCs, with negative results. As circulating B lymphocytes are in the G_0_ phase before their activation and beginning of clonal expansion, we postulated if *BMPR2* expression in these cells could be cell cycle phase dependent. After a literature review, we decided to perform the EBV immortalization, as the expression of the *BMPR2* in these cell lines had been previously described 7, 33, 34, 35.

We found increased expression levels in the patients with GD when compared against controls, and this overexpression may support the idea of BMPR2 being related to autoimmunity processes. Even though when we analysed the expression in the different cell cycle phases in each group, we did not find significant differences. This could be explained by the alterations produced by the EBV infection needed to produce the LCLs, which is able to alter the cell cycle check points 36. Increasing the number of samples could be necessary to obtain a significant expression pattern between the cell cycle phases.

In 2005, Roberts *et al*. speculated about the possibility of BMPR2 influencing in the development of thyroid pathologies after finding these kinds of diseases in 100% of the IPAH patients carrying *BMPR2* mutations, while only 14% of the non‐carriers of *BMPR2* mutations referred these pathologies 37. More recently, Satoh *et al*. demonstrated the existence of increased prevalence and predisposition to the development of thyroid autoimmune diseases in patients of APAH 38. This evolution towards autoimmunity might be based on the apparent relation between the BMPR2 and the differentiation and maturation of the T lymphocytes in the thymus 39, 40, where mutations in this gene may interfere in the normal development of thymocytes 39. This was also suggested to explain the presence of connective tissue diseases in APAH 41.

When we separated the GD patients group into carriers and non‐carriers of the variant, we found slightly increased values of *BMPR2* expression in the carriers, but not statistically significant. In addition, the protein expressed in the LCLs of both groups matched the predicted molecular weight of 115 kD. It would be necessary to analyse a great number of samples in order to confirm these expression differences.

The TGF‐β/BMP signalization pathway is one of the most important routes for cell stability making its presence ubiquitous within the organism. Its influence may vary between cell types because of the existing cell receptors and the context of hormones and growth factors 42, 43, but its main roles remain being the regulation of cell proliferation, differentiation, apoptosis and development. On the one hand, TGF‐β can appear in three isoforms (TGF‐β1, TGF‐β2 and TGF‐β3) with TGF‐β1 being the most studied 44. The TGF‐β1 binds to the TGF‐β type 2 receptor, and after its activation, it binds to the TGF‐β type 1 receptor and phosphorylates it in the serine/threonine residues. Then, by the union of two receptors of each type, an heterocomplex with activity activin‐like kinase is formed that will phosphorylate the Smad 2/3 proteins, which will then be able to translocate towards the nucleus to regulate gene expression 45. This gene regulative effect can be also caused without the need of phosphorylation by the activation of the Smad 4.

On the other hand, the BMP signalization starts when BMP binds to the BMPR2, which as a serine/threonine kinase protein phosphorylates one of the BMP type 1 receptors (ALK‐3/BMPR‐IA, ALK‐6/BMPR‐IB or ALK‐2) forming a heterocomplex 46. Upon its activation, the type 1 receptor activates and phosphorylates the Smad 1/5/8 proteins 47, which will then bind to the Smad 4 protein and translocate to the nucleus to regulate gene expression. The Smad 4 protein is therefore common in both pathways playing a central role. There has been reported that when BMPR2 signalization through the Smads proteins decrease, TGF‐β signalization increases, and this effect is the result of their competition for the usage of the Smad 4 protein 48. However, there is evidence of a more complex reciprocal regulation for both pathways 49. Thus, the increased *BMPR2* expression we found in our study could be paired with a decrease in the expression of the *TGF‐*β*1*, something that has been demonstrated for patients with GD in a recent study carried out in biopsy samples from thyroid pathology patients 50.

TGF‐β1 has been widely related to cell proliferation and tumour repression in thyroid healthy tissue, but it is believed to have the opposite function in cancerous thyroid 45, 51, 52, 53, 54. Thus, the different types of thyroid neoplasia show varying levels of the TGF‐β1. In our case, all the patients with GD presented goitre, five diffuse and two nodular, so we could hypothesize that the goitre could be caused by the dysregulation of the TGF‐β/BMP signalling pathway. BMPR2 overexpression should be confirmed in thyroid tissue. Although, the role of the TGF‐β1 pathway has been extensively analysed in the thyroid gland, scarce studies have referred to the role of BMPR2 in this tissue.

The unanswered question is still why this variant appears in high frequency in patients with GD. We could consider a founder effect as a plausible explanation. However, we could neither rule out the possibility of this variant acting as an enhancer, affecting gene expression of a completely unrelated gene located within the chromosome 2. This enhancer activity has been reported with molecular distances of up to 1 Mbp 55. Theoretically, a single base pair change as the reported in the c.419‐19delT could make the enhancer miss its target, altering gene expression and affecting carrier's phenotype.

Chromosome 2 has a wide variety of genes, but we would like to suggest a couple of candidates to be modulated by this possible enhancer. Because of its proximity to the *BMPR2* gene, the *CTLA4* gene and the *cluster of differentiation 28* (*CD28*; OMIM # 186760) would be suitable choices as they both are located in the q.33 position. *CTLA4* and *CD28* are homologous proteins that bind to the B7 proteins 56. *CTLA4* acts as a checkpoint in the immune response, transmitting an inhibitory signal to T cells and down‐regulating immune responses, whereas CD28 (UNIPROT #10747) plays the contrary role, transmitting a stimulatory signal 57. Also, *CTLA4* has already been widely linked with GD 31, 58, 59, 60, so the possibility of an enhancer modulating its expression or its counterpart's is interesting, as it could possibly alter GD development. A misbalance between *CTLA4* and *CD28* could mean that T cells would be activating B cells in a higher ratio, being the presence of anti‐TSHR antibodies the cause of GD, this augmented antibody production could affect GD development and evolution.

The main limitation of our study is the low number of samples included in the molecular analysis, as it diminishes our ability to draw robust conclusions. Besides, we could not perform a genotype–phenotype correlation to verify whether the c.419‐43delT variant or the differences in *BMPR2* genetic expression affect patients’ phenotype. However, the comprehensive genetic analysis performed and the strength of some findings add value to our results.

To conclude, the c.419‐19delT appears in high frequency in patients with GD, and it does not produce alterations in the splicing process in the tested cell lines, although it should be checked in thyroid and vascular endothelial cells due to the high variable behaviour of the spliceosome between tissues. The *BMPR2* gene is overexpressed in patients with GD, some articles referred decreased expression of TGF‐β1 in GD patients’ thyroid, and this could be explained by Smad 4 protein competition between the TGF‐β/BMP signalization routes. Thus, BMPR2 should be taken into consideration as a possible therapeutic target for GD novel treatments.

## Conflict of interest

The authors declare that they have no competing interests.
